# Metabolic Disorders and Steatosis in Patients with Chronic Hepatitis C: Metabolic Strategies for Antiviral Treatments

**DOI:** 10.1155/2012/264017

**Published:** 2012-06-04

**Authors:** Munechika Enjoji, Motoyuki Kohjima, Kazuhiro Kotoh, Makoto Nakamuta

**Affiliations:** ^1^Health Care Center, Fukuoka University, 8-19-1 Nanakuma, Jonan-ku, Fukuoka 814-0180, Japan; ^2^Clinical Research Center, Kyushu Medical Center, National Hospital Organization, Fukuoka 810-8563, Japan; ^3^Department of Gastroenterology, Kyushu Medical Center, National Hospital Organization, Fukuoka 810-8563, Japan; ^4^Department of Hepatology and Pancreatology, Kyushu University Hospital, Fukuoka 812-8582, Japan

## Abstract

It has been reported that hepatitis C virus (HCV) infection is closely associated with hepatic metabolic disorders. Hepatic steatosis and insulin resistance are both relatively common in patients with chronic hepatitis C. Recent investigations suggest that HCV infection changes the expression profile of lipid-metabolism-associated factors in the liver, conferring advantages to the life cycle of HCV. Moreover, insulin resistance and steatosis are independent predictors of impaired response to antiviral treatment in chronic hepatitis C. In this paper, we summarize our current knowledge of hepatic metabolic disorders and describe how HCV leads to and exploits these hepatic disorders. We also discuss the clinical significance of insulin sensitizers used to improve insulin resistance and lipid modulators used to manage lipid metabolism as potential treatment options for chronic hepatitis C.

## 1. Introduction

Hepatic steatosis is a well-documented histological characteristic of chronic hepatitis C virus (HCV) infection [1]. Insulin resistance or impaired glucose metabolism, is linked to hepatic steatosis in patients with chronic hepatitis C (CH-C). It is widely considered that hepatic steatosis in patients with CH-C is caused by lipid metabolic disorders, in which insulin resistance plays an important role [2]. Fat accumulation promotes oxidative stress and inflammatory reactions. A considerable number of studies have also suggested that various HCV proteins lead to alterations in lipid synthesis, catabolism and transport. In particular, HCV core protein was reported to contribute to these metabolic changes and induce reactive oxygen species generation [3, 4]. Clinically, hepatic steatosis and insulin resistance in CH-C patients are associated with hepatic fibrosis, an increased frequency of hepatocellular carcinoma, and a poor response to pegylated interferon (peg-IFN) plus ribavirin combination therapy [5].

## 2. HCV Infection and Insulin Resistance

It has been reported that hepatic steatosis is correlated with viral load; approximately 50% of patients with CH-C have hepatic steatosis, which enhances disease progression [6, 7]. Recent studies have shown that, as in nonalcoholic fatty liver disease (NAFLD), insulin resistance and an increased fatty acid supply to the liver are important pathogeneses of steatosis in CH-C [8]. In CH-C patients, the occurrence of insulin resistance is independent of visceral adipose tissue and hepatic steatosis and irrespective of the HCV genotype [9]. In our experience, insulin resistance is frequently observed in nonobese patients, and 36.8% patients with CH-C had a homeostasis model assessment-insulin resistance (HOMA-IR) index ≥2.5 [10]. Even though the association between the severity of insulin resistance and HCV viral load or genotype is controversial, viral eradication by antiviral therapy actually improves insulin sensitivity [11–13]. Despite the close association between chronic HCV infection and the presence of insulin resistance, the pathogenic basis of this interaction remains to be elucidated. Increasing epidemiological and experimental data suggest that the HCV core protein impairs insulin signaling, mostly by activating tumor necrosis factor *α* (TNF*α*) and members of the suppressor of cytokine signaling (SOCS) family [9, 14, 15].

SOCS proteins, which are induced by proinflammatory cytokines, induce proteasomal degradation of their target proteins, including insulin receptor substrate (IRS). Experimentally, upregulation of SOCS-1 and -3 in the liver leads to insulin resistance through several mechanisms, including degradation of IRS1 and IRS2 inhibition of insulin receptor kinase activity, and downregulation of IFN-associated innate immunity [16–18]. Activated TNF*α* inhibits tyrosine phosphorylation of IRS1 and IRS2, and impairs glucose transporter (GLUT)-4 translocation to the cell membrane, leading to insulin resistance and hyperinsulinemia, which can increase glycogenolysis and fatty acid synthesis [19, 20].

These changes may lead to hepatic steatosis by increasing the influx of free fatty acids via peripheral lipolysis, activation of lipogenesis-associated factors, reduced fatty acid oxidation, and decreased formation of very low-density lipoprotein (VLDL) [21]. IRS1 and IRS2 are closely linked to the regulation of glucokinase expression and lipogenic enzymes, such as sterol-regulatory element-binding protein 1c (SREBP-1c), respectively.

HCV infection, mainly through activity of the HCV core protein, decreases the expression and activity of peroxisome proliferator-activating receptor (PPAR)-*α*/*γ* in hepatocytes [22]. These effects may constitute strategies for viral survival and proliferation. PPAR*α* and PPAR*γ* transcriptionally regulate fatty acid *β*-oxidation and insulin sensitivity, respectively. Indeed, PPAR*γ* agonists, thiazolidinediones, improve insulin sensitivity in CH-C patients. In our earlier study, we found that telmisartan, an angiotensin II receptor blocker and a potential partial PPAR*γ* agonist, had significant therapeutic effects by attenuating insulin resistance and liver injury in patients with CH-C [10].

## 3. Lipid Metabolic Disorders in HCV-Infected Liver

**Figure 1:**
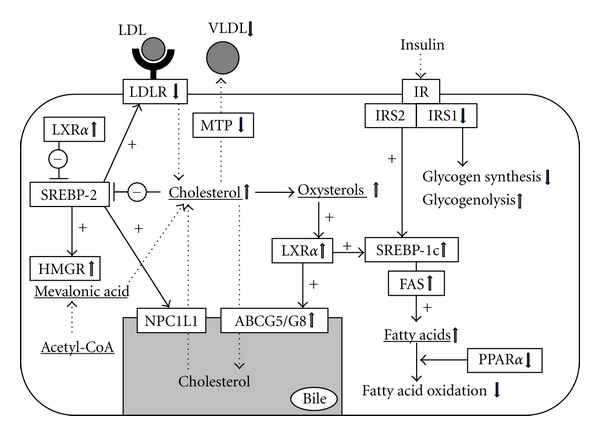
Expression profile of lipid metabolism-associated factors in chronic hepatitis C. ABCG5/G8, ATP-binding cassette G5/G8; FAS, fatty acid synthase; HMGR, HMG-CoA reductase; IR, insulin receptor; IRS, insulin receptor substrate; LDLR, LDL receptor; LXR*α*, liver X receptor *α*; MTP, microsomal triglyceride transfer protein; NPC1L1, Niemann-Pick C1-like 1; PPAR*α*, peroxisome proliferator-activating receptor *α*; SREBP, sterol regulatory element-binding protein.

A close association between HCV infection and lipid metabolism has been reported, and host metabolic factors and viral factors are likely to be involved in the pathogenesis of hepatic steatosis (see Figure 1). HCV core protein, which is localized to the membrane of lipid vesicles, induces hepatic fat accumulation by activating SREBP-1c [23, 24]. It also inhibits microsomal triglyceride transfer protein (MTP) activity, which is needed for VLDL assembly and excretion [25]. HCV infection decreases hepatic expression of PPAR*α*, which negatively regulates fatty acid uptake and positively regulates *β*-oxidation, and promotes *de novo* lipid and cholesterol generation by enhancing the activities of SREBP-1 and -2 [24, 26].

In our evaluation of the expression of lipid metabolism-associated genes, the regulation of lipid metabolism was impaired in HCV-infected liver [27, 28]. The expression profiles revealed that HCV infection induced intrahepatic accumulation of cholesterol as well as triglycerides, resulting in a marked reduction of low-density lipoprotein receptor (LDLR) to decrease LDL-cholesterol uptake, and upregulated ATP-binding cassette G5 to increase cholesterol output. However, *de novo *cholesterol and fatty acid synthesis continued to increase, perhaps because of disrupted negative feedback pathways. This uncontrolled expression pattern is almost similar in NAFLD [29, 30]. However, HCV core protein interferes with the assembly and secretion of VLDL via inactivation of MTP, leading to hypobetalipoproteinemia, whereas, in NAFLD, MTP activity is enhanced and hyperlipidemia is common [8]. This expression pattern was also apparent in a preliminary evaluation of an HCV replicon system. Cholesterol is synthesized in hepatocytes through the mevalonate pathway, which is promoted by several enzymes, including HMG-CoA reductase (HMGR). Normally, the expression of LDLR and HMGR is regulated by the transcription factor SREBP-2 according to the intracellular cholesterol load. However, despite marked cholesterol accumulation, HMGR expression was greatly enhanced in HCV-infected liver [27, 28]. During cholesterol overload, the levels of cholesterol metabolites increase, including oxysterols, which act as agonistic ligands of liver X receptor-*α* (LXR*α*). These metabolites activate the LXR*α*-SREBP1c axis, which ultimately leads to activation of fatty acid synthesis. Notably, LXR*α* expression was also enhanced in HCV-infected liver [27, 28].

In addition to the core and nonstructural HCV proteins, the activation of cholesterol and fatty acid biosynthesis play a critical role in viral assembly, release, and infectivity [31–33]. Accordingly, viral interactions with the host's lipid metabolic pathways appear to be essential for the life cycle of HCV. Attachment of the virus to the cell surface LDLR represents the first stage of HCV entry into hepatocytes, and *β*-lipoproteins influence HCV proliferation [34, 35]. Serum HCV antigen levels are negatively correlated with serum *β*-lipoprotein levels [36]. The resulting lipid droplets supply lipoproteins and lipids and provide a site for viral assembly. These changes seem to be necessary or beneficial for HCV replication. The mevalonate pathway of *de novo* cholesterol synthesis, in which HMGR is a rate-limiting enzyme, is also responsible for the synthesis of farnesyl pyrophosphate and geranylgeranyl pyrophosphate that are essential for viral replication [37]. These molecules are needed to activate small GTPases, such as Rho and Ras. Therefore, HCV may need lipids not only as components of virus particles but also to modulate the host's cell signaling pathways.

## 4. Role of the Cannabinoid System in HCV-Infected Liver

Endocannabinoids, such as anandamide and 2-arachidonoylglycerol (2-AG), are synthesized from lipid precursors in cellular membranes and specifically target cannabinoid receptors (CB) 1 and CB2 [38]. Recent studies have suggested that the hepatic cannabinoid system is involved in the pathogenesis of NAFLD by activating CB1 and that steatogenic factors, such as a high-fat diet, induce the synthesis of endocannabinoids and CB1 [39–41]. Although the signal transduction pathways have not been fully characterized, CB1 activation enhances the expression of several lipogenic factors, including SREBP-1c and fatty acid synthase (FAS) and downregulates factors involved in fatty acid oxidation, such as carnitine palmitoyltransferase I, resulting in steatosis and insulin resistance. Experimentally, steatogenic factors appear to activate CB2, but CB2 is mostly found in immune system cells. Therefore, CB2 activation may play a protective role against the inflammatory and fibrogenic responses in steatohepatitis [42, 43].

Because daily cannabis use was proposed as a risk factor for the severity of steatosis and progression of fibrosis in patients with CH-C [44], we determined the role of the hepatic cannabinoid system in HCV infection using HCV subgenomic replicon cells, which stably express viral nonstructural proteins (NS3, NS4A/4B, NS5A, and NS5B). Although the tested cannabinoid, anandamide, cannot be detected in culture medium, CB1 expression and triglyceride accumulation increased in replicon cells, as did the expression levels of several lipid synthesis-associated genes (SREBP-1c, FAS, and HMGR). IFN*α* treatment downregulates the expression of viral proteins and reduces triglyceride accumulation and gene expression of CB1, SREBP-1c, FAS, and HMGR. Meanwhile, treatment with a CB1 agonist increased, and a CB1 antagonist treatment decreased triglyceride accumulation in replicon cells.

These findings support the possibility that HCV infection activates the hepatic cannabinoid system and enhances steatotic changes in the liver. In healthy human liver, the hepatocytic expression of CB1 and CB2 is very low or even absent, as are endocannabinoid levels [45–48]. Marked upregulation of these receptors and endocannabinoid levels (anandamide and 2-AG) has been reported in the cirrhotic liver [45, 49–51]. Moreover, in acute liver damage, the expression of CB1 and CB2 is enhanced, and the degree and duration of inflammation may be an important factor for controlling CB1 expression or activation of the cannabinoid system. However, in a quantification assay using real-time polymerase chain reaction, CB1 gene expression was very low in liver samples from CH-C patients and healthy individuals (unpublished data). In patients with CH-C, more severe inflammation or fibrosis may be needed to activate the cannabinoid system.

As described above, there is a discrepancy between *in vitro *data in HCV replicon cells and findings in the liver of patients with CH-C. Therefore, it is questionable whether activation of the cannabinoid system significantly affects metabolic disorders in the HCV-infected liver. Of note, some researchers have proposed the existence of cannabinoid receptors other than CB1 and CB2 and endocannabinoids other than anandamide and 2-AG [52], although these have not yet been clearly detected. Therefore, still unknown cannabinoids and/or receptors may play a leading role in hepatic metabolic disorders in patients with CH-C.

## 5. Therapeutic Strategies Using Metabolic Modulators

Clinically, antioxidants, such as ursodeoxycholic acid and vitamin E, have been commonly used for NAFLD and CH-C patients as a liver supporting therapy. In many patients with insulin resistance, insulin sensitizers, such as metformin and thiazolidinediones, have shown improving effect of liver biochemistry. Nowadays, IFN-based radical antiviral treatments are generally accepted for patients with CH-C. Hepatic steatosis and insulin resistance are negative predictors for sustained virological response (SVR) in patients with CH-C treated with peg-IFN*α* plus ribavirin combination therapy [53, 54]. In recent meta-analyses, HOMA-IR, a marker of insulin resistance, is negatively correlated with SVR, irrespective of viral genotype [55, 56]. Therefore, lifestyle modifications, such as weight reduction by exercise and nutritional management, are recommended to enhance the effects of antiviral treatments. Moreover, it is justifiable that the use of agents targeting insulin resistance and dyslipidemia can improve the SVR rate achieved with IFN-based antiviral treatments.

Insulin sensitizers, metformin and thiazolidinediones, may increase the response to antiviral treatments [57]. In a recent clinical trial of insulin-resistant patients with CH-C genotype 1, adding metformin to standard peg-IFN*α* plus ribavirin therapy improved insulin sensitivity. Metformin also tended to improve SVR, particularly in females, although a statistically significant difference was not seen compared with patients receiving placebo [58]. Meanwhile, the effects of pioglitazone on SVR in patients with CH-C and insulin resistance are controversial. Pioglitazone combined with peg-IFN*α* plus ribavirin therapy was used as retreatment and in treatment-naïve patients, but results of two pilot trials were disappointing [59, 60]. However, some reports have described that the addition of pioglitazone to standard therapy improves SVR and insulin sensitivity [61, 62]. This discrepancy may be explained, at least in part, by genotype dependency and host characteristics.

As described above, the synthesis of cholesterol and fatty acids is still activated in the liver of patients with CH-C, despite lipid overaccumulation. Therefore, correcting cholesterol and fatty acid synthesis by lipid modulators may help to reduce steatosis and improve SVR with antiviral treatments. Considering that cholesterol synthesis is enhanced in HCV-infected liver, it is plausible that HMGR inhibitors (statins) could have antiviral effects, because statins were recently reported to suppress HCV replication [63]. In fact, it was reported that statins do impede HCV replication by inhibiting host protein geranylgeranylation, and FBL2 has been identified as a geranylgeranylated cellular protein required for HCV RNA replication [64]. Retrospective analyses of patients with CH-C treated with peg-IFN plus ribavirin combination therapy have shown that serum cholesterol and the use of statins are positive predictors of SVR [65, 66]. In clinical trials, SVR was improved by adding fluvastatin or pitavastatin to peg-IFN plus ribavirin treatment [67–69]. Although antiviral activity has been experimentally demonstrated in most statins without pravastatin [63], a statin with a more activity may achieve better SVR rates. Of note, protease inhibitors, such as telaprevir, and statins taken together may raise the blood levels of statins and increase the risk of myopathy, kidney damage, and kidney failure. It was also reported that polyunsaturated fatty acids (PUFAs) inhibit HCV replication by a still unclear mechanism, independent of their roles in regulating lipogenesis and that eicosapentaenoic acid (EPA), an n-3 PUFA, inhibits HCV replication in the replicon system and suppresses SREBP-1c activity [70–72]. Additionally, administration of EPA allows maintenance of the original ribavirin dose in patients with CH-C during peg-IFN plus ribavirin combination therapy [73]. Using HCV replicon systems, it was reported that statins and EPA have suppressive effects against HCV replication and synergistic antiviral action with IFN [37, 70, 71, 74, 75].

Based on experimental and therapeutic evidence, concomitant administration of a statin and EPA with peg-IFN plus ribavirin therapy is pathophysiologically promising for patients with CH-C. Accordingly, we are now performing a clinical trial using a new antiviral strategy for patients with CH-C genotype 1b in which pitavastatin (2 mg/day) and EPA (1,800 mg/day) are added to standard peg-IFN plus ribavirin therapy. According to recent clinical studies of patients with CH-C genotype-1b, mutation of amino acids 70 and 91 in the core region of HCV-1b, as a virus-related factor, and genomic variation of the *IL28B* gene (rs8099917), as a host-related factor, are strong predictors of the outcome of peg-IFN plus ribavirin combination therapy [76–79]. Within the core protein, substitution of amino acid 70 seems to be more influential on the outcome than substitution of amino acid 91 [79–81]. At present, our trial has yielded several important findings (unpublished data). First, add-on pitavastatin and EPA therapy conferred significantly higher SVR rates than did standard therapy. Second, add-on therapy significantly improved SVR rates, particularly in patients with the minor variant (TG + GG) of *IL28B* (rs8099917), in whom SVR is expected to be poor. Of note, among patients treated with add-on therapy, genomic variation of *IL28B* still predicts clinical outcomes, because SVR rates were significantly higher in patients with the major variant (TT) than in those with minor variants. Third, mutation of core amino acid 70, which is a strong negative predictor of SVR in standard peg-IFN plus ribavirin therapy, did not diminish the outcomes of add-on therapy.

## 6. Conclusions

Steatosis and insulin resistance induced by HCV infection are, at least in part, critical factors for the progression of CH-C and can influence the outcome of antiviral treatments. Therefore, managing these metabolic disorders by administering insulin sensitizers and lipid modulators has been examined to increase the therapeutic response of standard treatments. In particular, concomitant treatment with pitavastatin and EPA may achieve considerable improvements in the efficacy of peg-IFN plus ribavirin combination therapy, particularly in patients with CH-C resistant to standard peg-IFN plus ribavirin therapy.

##  Conflict of Interests

The authors have no conflict of interests to declare.
